# Spatial variations in the molecular diversity of dissolved organic matter in water moving through a boreal forest in eastern Finland

**DOI:** 10.1038/srep42102

**Published:** 2017-02-10

**Authors:** Jun’ichiro Ide, Mizue Ohashi, Katsutoshi Takahashi, Yuko Sugiyama, Sirpa Piirainen, Pirkko Kortelainen, Nobuhide Fujitake, Keitaro Yamase, Nobuhito Ohte, Mina Moritani, Miyako Hara, Leena Finér

**Affiliations:** 1 Institute of Decision Science for a Sustainable Society, Kyushu University, Fukuoka 811-2415, Japan; 2 Graduate School of Human Science and Environment, University of Hyogo, Hyogo 670-0092, Japan; 3 National Institute of Advanced Industrial Science and Technology, Tsukuba 305-8568, Japan; 4 Natural Resources Institute Finland, P.O. Box 68, FIN-80101 Joensuu, Finland; 5 Finnish Environment Institute, P.O. Box 140, FIN-00251 Helsinki, Finland; 6 Faculty of Agriculture, Kobe University, Kobe 657-0013, Japan; 7 Hyogo Prefectural Technology Center for Agriculture, Forestry and Fisheries, Forestry and Forest Products Research Institute, Hyogo 671-2515, Japan; 8 Department of Social Informatics, Graduate School of Informatics, Kyoto University, Kyoto 606-8501, Japan

## Abstract

Dissolved organic matter (DOM) strongly affects water quality within boreal forest ecosystems. However, how the quality of DOM itself changes spatially is not well understood. In this study, to examine how the diversity of DOM molecules varies in water moving through a boreal forest, the number of DOM molecules in different water samples, i.e., rainwater, throughfall, soil water, groundwater, and stream water was determined using Fourier transform ion cyclotron resonance mass spectrometry (FT-ICR MS) in Norway spruce and Scots pine stands in eastern Finland during May and June 2010. The number of molecular compounds identified by FT-ICR MS (molecular diversity) ranged from 865 to 2,194, revealing large DOM molecular diversity in the water samples. Additionally, some of the molecular compounds were shared between different water samples. The DOM molecular diversity linearly correlated with the number of low-biodegradable molecules, such as, lignin-like molecules (lignins), but not with dissolved organic carbon concentration. The number of lignins shared between different sampling locations was larger than that of any other biomolecular class. Our results suggest that low-biodegradable molecules, especially lignins, regulate spatial variations in DOM molecular diversity in boreal forests.

Dissolved organic matter (DOM) plays a central role in changes in water chemistry within boreal forest ecosystems. It affects the acidity and color of the water that passes through the tree canopy or forest floor^1^,^2^,^3^,^4^,^5^. It forms complexes with many nutrients and metals and transports them from forest ecosystems to watercourses^6^,^7^,^8^,^9^,^10^. Additionally, changes in the quality and quantity of DOM influence ecological processes, because DOM is a source of energy and is easily modified by microorganisms^11^,^12^.

Boreal forests contain more than one third of the carbon retained in forest vegetation and soils globally^13^. According to a recent inventory of organic carbon pools in boreal forest soils in Fennoscandia, 70–80% of the organic carbon is found in the upper 100 cm of mineral soil^14^. Previous studies showed that dissolved organic carbon (DOC) concentration was five times higher in throughfall than in bulk deposition and also five times higher in soil water in the O-horizon than in throughfall^15^,^16^. This is because DOM leaches into the water from plant tissues and dead organic matter, and is also microbially decomposed in soils. Additionally, DOM can be retained in mineral soils since humic substances are associated with amorphous iron and aluminum^17^. The release and retention of DOM by biogeochemical processes should alter not only the amount of DOM but also its composition. Hydrophobic DOM is preferentially adsorbed by forest soil particles, which could increase the relative abundance of hydrophilic compounds in soil water^18^. An increase in DOC concentration in soil water after clear-cutting was also reported, suggesting that forestry operations affect the chemical composition of DOM^19^. Considering the results reported by Bourbonniere and Creed^20^ that fresher and younger forest-floor materials released more biodegradable DOM than older ones, it is possible that DOM released from logging residues or litter in newly-planted forest stands has a labile property. However, few studies have focused on how the chemical composition of DOM changes in water as it moves through forest ecosystems^21^.

Recent progress in the chemical characterization of DOM has enabled us to obtain an overall picture of molecular alterations/transitions of DOM in water collected from forests. Fourier transform ion cyclotron resonance mass spectrometry (FT-ICR MS) is one of the most innovative methods and allows us to detect different types of molecules in DOM based on the cyclotron frequency of charged ions in a magnetic field. This method has provided reproducible and reliable data concerning the mass spectrum of DOM in river and marine water samples, which consist of more than a hundred different types of DOM molecules^22^,^23^,^24^,^25^. In addition to information on the number and types of DOM molecules, the van Krevelen diagram^26^ enables us to divide each molecule detected by FT-ICR MS into different biomolecular classes, such as lignin-like molecules, proteins, and tannin-like molecules, for descriptive purposes^27^,^28^. Detailed information on DOM might help us understand the processes driving changes in the chemical composition of DOM and the factors controlling them. Kalbitz *et al*.^29^ investigated changes in the chemical composition of DOM samples extracted from different materials, including maize straw, forest floors, peat, and agricultural soils, during a 90-day incubation using several methods, such as ultraviolet absorbance, fluorescence emission spectroscopy, and Fourier transform infrared spectroscopy. Their results indicated that the partial degradation of higher molecular weight compounds, such as lignin dimers and alkylaromatics, increases the proportion of lower molecular weight compounds, such as lignin monomers and phenols, which are biodegradation products. They also indicate that DOM biodegradation can result in relative enrichment of aromatic compounds, which are relatively stable and slowly mineralized. These results suggest that chemical composition of DOM varies mainly via biotic processes, whereas there are molecular compounds that are more stable and move along with the water through the ecosystem. It is expected that the FT-ICR MS analysis and subsequent biomolecuar classification enhance our understanding not only of chemical aspects but also of biotic processes in the molecular alteration/transition of DOM associated with the water movement.

The objectives of this study were (1) to evaluate variations in molecular diversity and the molecular compounds divided into specific biomolecular classes in rainwater passing through a boreal forest using FT-ICR MS and van Krevelen diagrams, and (2) to examine how sampling location affects DOM molecular compounds in a boreal forest catchment in eastern Finland.

Our hypotheses were as follows:

1. The number of molecular compounds detectable by FT-ICR MS (i.e., molecular diversity) and subsequently the number of each biomolecular class vary with water movement (i.e., rainwater to stream water via throughfall, soil water, and groundwater).
2. The molecular compounds identified as the same DOM compound exist at different sampling locations.
3. DOM molecular diversity increases with increasing DOC concentration.

## Results

### Molecular diversity and biomolecule classification of DOM

**Figure 1:**
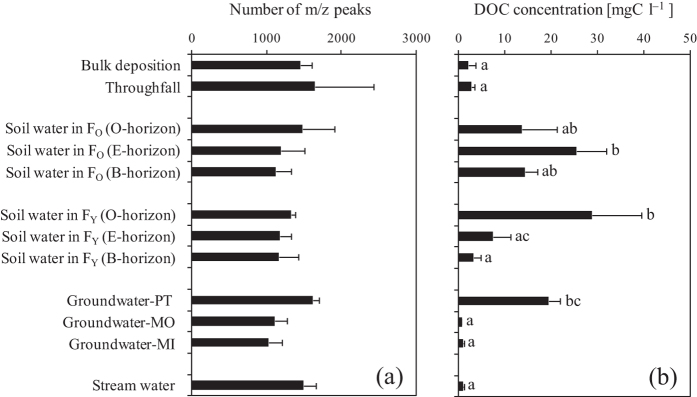
(**a**) Average values for the number of m/z peaks and (**b**) DOC concentration in bulk deposition, throughfall, soil water, groundwater, and stream water during the study period. Error bars represent standard deviations. Values with the same letter indicate no significant difference (*p* > 0.05). F_O_ and F_Y_ represent old-growth forest and young plantation stands, respectively. See Methods for the abbreviations of groundwater.

The number of DOM molecular compounds detected as m/z peaks (molecular diversity) was 1,293 ± 307 SD on average across all of the water samples and ranged from 865 to 2,194 (Fig. 1a). The coefficient of variation (CV) for the molecular diversity was 23%. The molecular diversity did not differ significantly among sampling locations (*p* > 0.05).

**Figure 2:**
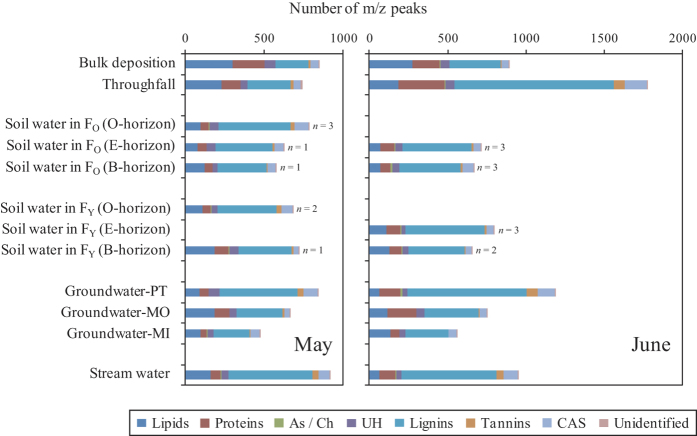
Classification of the m/z peaks into specific biomolecular classes using a van Krevelen diagram. F_O_ and F_Y_ represent old-growth forest and young plantation stands, respectively. *n* values represent the number of lysimeters which collected soil water. The average values are shown for soil water samples with *n* = 2 or *n* = 3. See Methods for the abbreviations of groundwater and biomolecular classes and see Figures S1 and S2 for variations in the number of m/z peaks for each biomolecular class in soil water samples.

Each DOM molecular compound was assigned to one of seven biomolecular classes (i.e., lipids, proteins, aminosugars/carbohydrates (As/Ch), unsaturated hydrocarbons (UH), lignin-like molecules (lignins), tannin-like molecules (tannins), or condensed aromatic structures (CAS)), or it was noted that it could not be assigned to any class. Our results revealed that the number of lignins was larger than that of any other class and only a few molecules of As/Ch were found in every sample except the bulk deposition sample obtained in May (Fig. 2). The number of lignins was especially large in throughfall, groundwater-PT, and stream water samples in June. The numbers of lipids or proteins were the second largest after lignins in the majority of the water samples; however, in some samples, the number of CAS was second to lignins.

### Relationships of biomolecular classes and DOC concentration to molecular diversity

**Figure 3:**
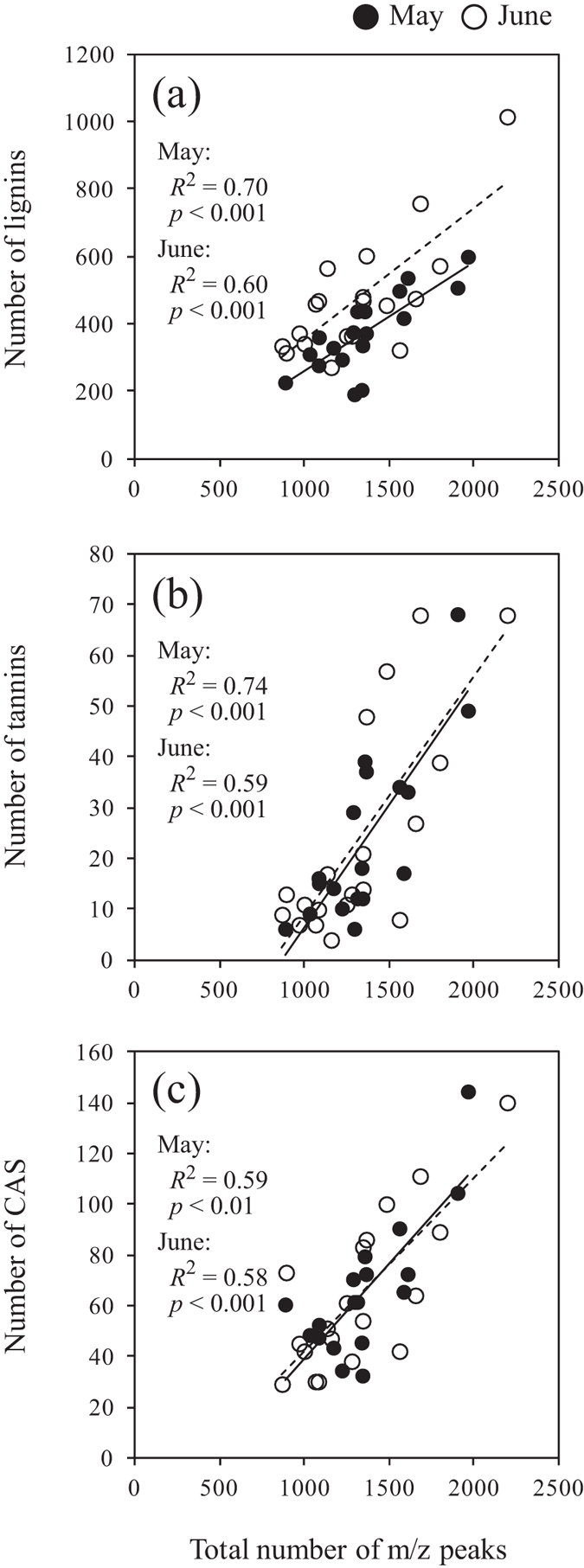
(**a**) Relationship between the total number of m/z peaks (molecular diversity) and the number of peaks representing lignin-like molecules (lignins), (**b**) tannin-like molecules (tannins), and (**c**) condensed aromatic structures (CAS). Solid and broken lines represent regression lines for data obtained in May and June, respectively.

DOM molecular diversity increased significantly with increasing numbers of lignins, tannins, and CAS in both May and June (Fig. 3a: *r* = 0.83, *p* < 0.001 in May, *r* = 0.77, *p* < 0.001 in June; 3b: *r* = 0.86, *p* < 0.001 in May, *r* = 0.77, *p* < 0.001 in June; 3c: *r* = 0.77, *p* < 0.01 in May, *r* = 0.76, *p* < 0.001 in June). The proportion of the sum of lignins, tannins, and CAS in the molecular diversity in soil water was generally high (28–56%) compared with those in bulk deposition and throughfall (20–31%), although not in comparison to the throughfall in June (56%).

**Figure 4:**
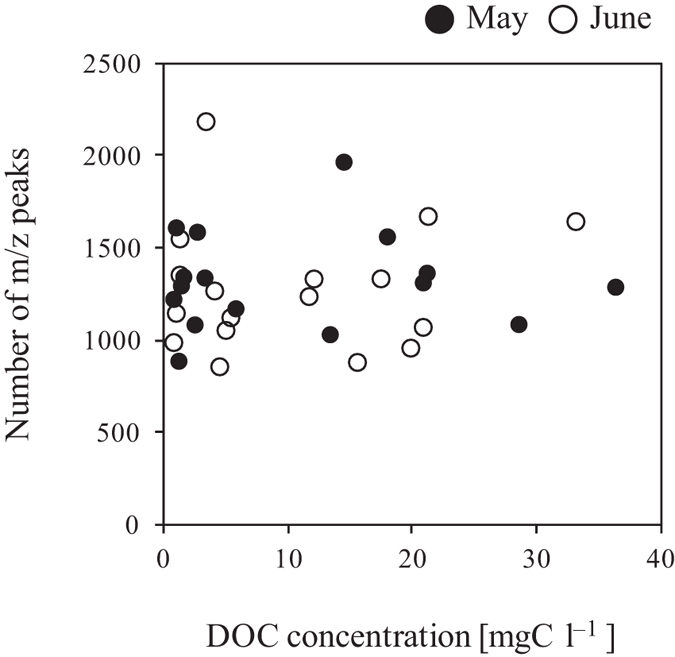
Relationship between the number of m/z peaks (molecular diversity) and DOC concentration.

The DOC concentration across all of the water samples was 11.2 ± 10.4 mgC l^–1^ on average, ranging from 0.7 to 36.4 mgC l^–1^ (Fig. 1b). The CV for DOC concentration was 93%. The DOC concentration differed significantly among the sampling locations (Fig. 1b: *p* < 0.01). There was no correlation between DOC concentration and DOM molecular diversity (Fig. 4: *r* = 0.09, *p* = 0.742 in May, *r* = 0.09, *p* = 0.722 in June).

### Spatial variation in DOM molecular composition

The number of molecular compounds detected as the same m/z peak between different water samples ranged from 153 to 1010. The proportion of those compounds in the molecular diversity ranged from 10% to 60% depending on sampling location. Additionally, the number of lignins shared between different water samples was larger than that of any other biomolecular class and ranged from 52 to 445.

**Figure 5:**
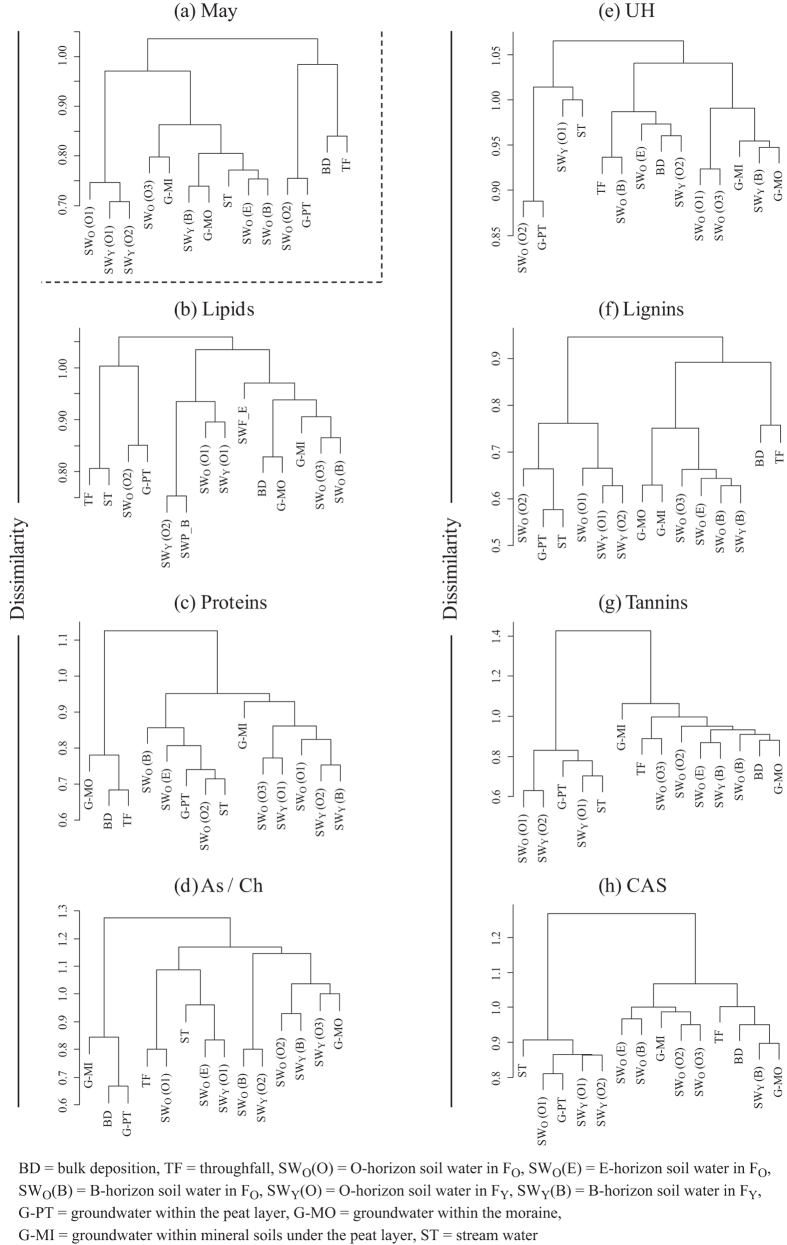
(**a**) Cluster dendrograms of different sampling locations for all molecular compounds identified and (**b**–**h**) for the seven biomolecular classes in May. The figure in the parenthesis represents the identification number of the three lysimeters (L1–L3). See Methods for the abbreviations of biomolecular classes.

**Figure 6:**
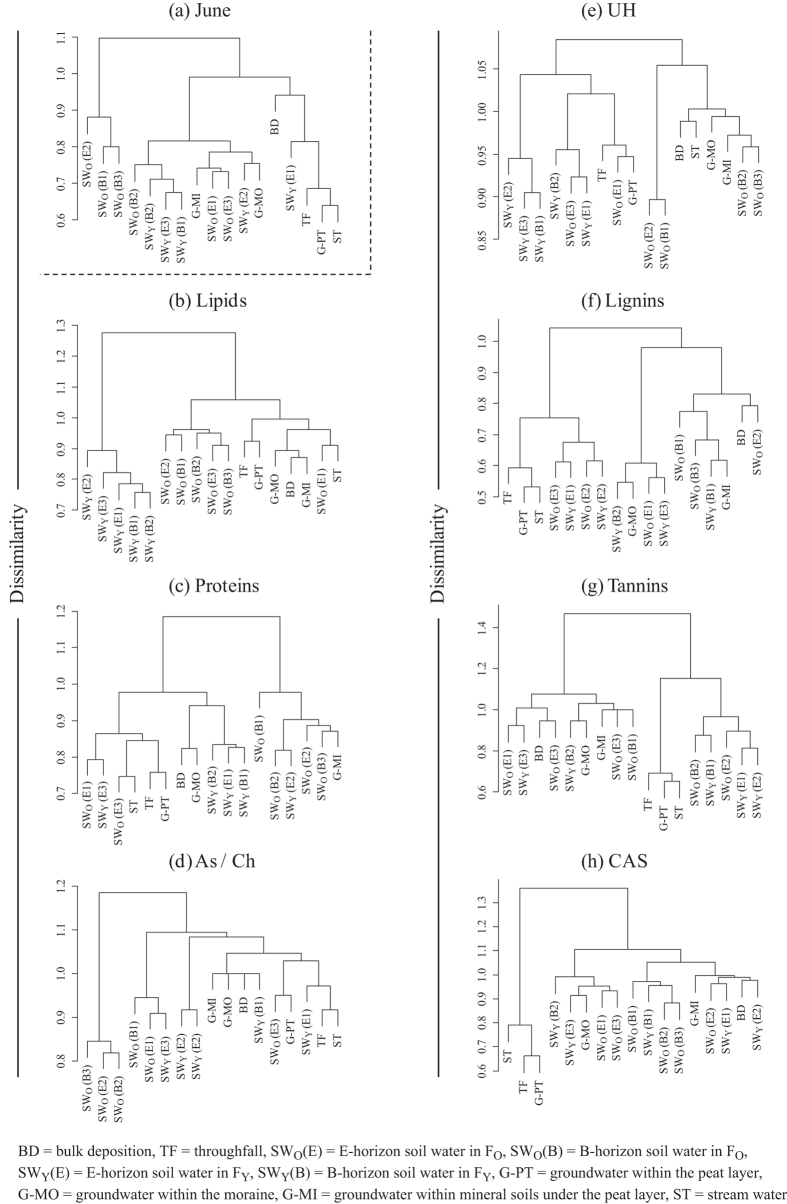
(**a**) Cluster dendrograms of different sampling locations for all molecular compounds identified and (**b**–**h**) for the seven biomolecular classes in June. The figure in the parenthesis represents the identification number of the three lysimeters (L1–L3). See Methods for the abbreviations of biomolecular classes.

**Figure 7:**
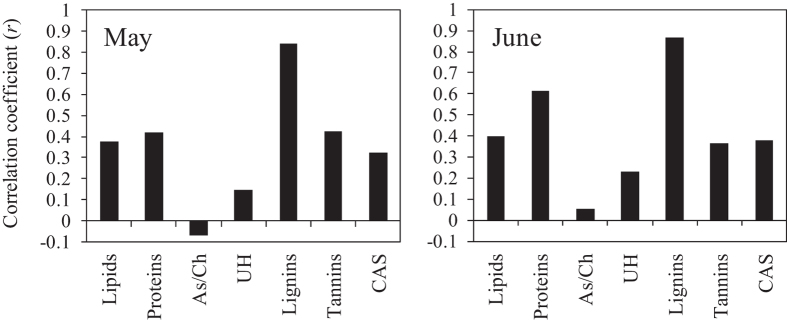
Correlation coefficients between cophenetic matrices of Jaccard’s distance for all molecular compounds identified and for each of the seven biomolecular classes in May and June. See Methods for the abbreviations of biomolecular classes.

The dendrogram including all species of molecular compounds detected by FT-ICR MS in May (Fig. 5a) showed that bulk deposition and throughfall formed one small cluster, indicating that these samples in May had many molecular compounds in common. The dendrograms for the seven biomolecular classes in May (Fig. 5b–h) also showed that lignins detected in the bulk deposition and throughfall formed one small cluster. O-horizon soil water (SW_O_(O1), SW_Y_(O1) and SW_Y_(O2) in Fig. 5), groundwater-PT and stream water were grouped into the same cluster in the lignins, tannins and CAS dendrograms in May (Fig. 5f–h). The bulk deposition and throughfall did not form one small cluster in the dendrogram including all molecular compounds identified in June (Fig. 6a); however, they were grouped into the same cluster when the dendrogram was cut at the dissimilarity level of 0.95. On the other hand, the dendrograms for the seven biomolecular classes in June (Fig. 6b–h) showed that liginins detected in the bulk deposition and throughfall were separated into the different clusters. Throughfall, groundwater-PT and stream water formed one cluster in the lignins, tannins and CAS dendrograms (Fig. 6f–h), which exhibited similar patterns to the dendrogram including all molecular compounds identified in June. Strong positive correlations were found between the cophenetic matrices of Jaccard’s distance for all molecular compounds and for lignins both in May and June (Fig. 7: *r* = 0.84, *p* < 0.001 in May, *r* = 0.87, *p* < 0.001 in June).

## Discussion

The results of the FT-ICR MS analysis revealed that thousands of different DOM molecules existed in each water sample collected from our study site, i.e., Kangasvaara catchment (Fig. 1a). FT-ICR MS is one of the most advanced instruments available for the detection of ionizable DOM because it has ultrahigh mass resolution and is often coupled with a non-destructive ion source, i.e., electrospray ionization (ESI)^30^. Kim *et al*.^31^ analyzed the molecular composition of DOM in a black-water stream located in the Pinelands of New Jersey, USA using FT-ICR MS and detected 18 peaks that can be assigned molecular formulas in the range of m/z 469.0–469.3. Additionally, Witt *et al*.^32^ investigated the molecular composition of fulvic acid in a water sample collected from the Suwannee River, USA, using FT-ICR MS and detected 21 peaks approximately at the m/z 411 within 0.35 in the mass spectrum. These previous studies indicated that FT-ICR MS allows for the detection of DOM molecular compounds within a narrow range of m/z values. Kim *et al*.^33^ detected over 3,000 molecular compounds in the mass spectrum of the water samples obtained in tropical and temperate forested streams. Furthermore, Wozniak *et al*.^34^ detected over 3,000 molecular compounds in aerosol-derived water-soluble organic carbon collected from rural areas in Virginia and New York, USA. Mazzoleni *et al*.^35^ detected 1,368 molecular compounds in water-soluble atmospheric organic matter contained in fog water. The number of molecular compounds detected as m/z peaks (molecular diversity) in this study was comparable to those observed in the previous studies. On the other hand, no clear trend in molecular diversity according to sampling location was observed, although the molecular compounds could be divided into biomolecular classes, in which the number of lignins was largest (Fig. 2) (hypothesis 1).

The linear relationships between DOM molecular diversity and the numbers of lignins, tannins, and CAS (Fig. 3) suggest that low-biodegradable molecules regulate changes in DOM molecular diversity^36^,^37^. Biodegradable molecules, such as hydrophilic organic matter leached from trees, are preferentially utilized by microorganisms, resulting in an abundance of low-biodegradable molecules, such as hydrophobic aromatic compounds remaining in soils^29^,^38^. Hur *et al*.^39^ suggested that microbial utilization of labile components, such as simple carbohydrates and amino acids, produced humic-like aromatic components in the DOM extracted from leaf litter. These would also be reflected in the generally higher proportion of the sum of lignins, tannins, and CAS in the molecular diversity in soil water than in bulk deposition and throughfall. However, some low-biodegradable molecules can be preferentially adsorbed by soil particles^18^,^40^. This might be reflected in the variations in the number of low-biodegradable molecules among replicates of soil water samples (see Figs S1 and S2). Similar patterns were found among the lignins, tannins and CAS dendrograms (Figs 6 and 7), implying that dynamics of low-biodegradable molecules were similar within boreal forest ecosystems.

Comparisons of the m/z value between different water samples revealed that common molecular compounds existed at different sampling locations (hypothesis 2). We found that the number of lignins shared between different sampling locations was larger than that of any other biomolecular class. Additionally, correlation analyses between the cophenetic matrices of Jaccard’s distance (Fig. 7) showed that the pattern of the degree of similarity among sampling locations for lignins reflects that for all molecular compounds detected by FT-ICR MS. Indeed, similar patterns were found between the dendrograms for lignins and all molecular compounds in May and June (Figs 5 and 6). These results indicate that lignins could regulate spatial variations in the number of the common molecular compounds. Lignins are phenolic polymers that originate mainly from vascular plants in terrestrial ecosystems and are considered to be recalcitrant organic matters because they are degraded at a lower rate than other substances originating from plants, such as, cellulosic and non-cellulosic polysaccharides and proteins^41^,^42^. This presumably allows lignins to exist as the molecular compounds common to different sampling locations in our study site.

On the other hand, a number of different molecular compounds existed between sampling locations since common molecular compounds accounted for less than 50% of the molecular diversity in the majority of the water samples. This may be related to the fact that the quality of DOM is altered by several processes. For instance, tree canopies and forest soils release DOM molecular compounds into rainwater; whereas in rainwater, they are microbially converted to lower molecular-weight compounds and/or some are retained in mineral soils via co-precipitation^40^,^43^,^44^,^45^. Sleighter and Hatcher^46^ noted that chemical structures could differ even though the assigned molecular formulas are the same between different water samples for the FT-ICR MS analysis. They also showed different chemical structures for C_26_H_32_O_11_ as an example of isomers, which represent two major refractory substances, i.e., lignin and carboxyl rich alicyclic molecule, and explained that these substances have different reactivities and sources though they have the same molecular formula. Therefore, it is possible that even molecular compounds considered lignins in the van Krevelen diagram were misidentified and consequently a larger number of different molecular compounds existed between sampling locations than observed. This also would be supported by Reetsma^47^ who states that the molecule plotted into the region of one of the biomolecular classes in the diagram does not necessarily belong to that class because the O/C and H/C ratios are insufficient criteria to ascribe a molecule to a certain biomolecuar class. However, several studies described that lignins are major components of refractory DOM in the terrestrial freshwater^12^,^30^,^48^. In forests, litter is considered to be an important source of soil DOM^49^ and especially coniferous litter, such as spruce and pine needle litter could release lignin-derived DOM components with a high aromaticity into the soils via microbial decomposition^50^. These support our conclusion that lignins could regulate spatial variations in the number of common molecular compounds and consequently molecular diversity in boreal forests.

Vertical profiles in DOM molecular diversity and DOC concentration within the Kangasvaara catchment (Fig. 1) revealed that variations in DOM molecular diversity were much smaller than those in DOC concentration. Presumably reflecting this result, molecular diversity did not change in association with DOC concentration (Fig. 4) (hypothesis 3). These results demonstrate that DOC concentration alone does not sufficiently explain spatial variations in molecular diversity. This could be attributed to the fact that molecular compositions detected by FT-ICR MS do not provide quantitative information on the molecular compounds present in DOM^51^. Alternatively, it could be attributed to the fact that not all DOM molecular compounds in water samples are detectable because the enrichment procedure of DOM from water samples, such as the C_18_ solid phase extraction method used, is incomplete^47^ and also because non-ionizable organic compounds are not characterized by FT-ICR MS^30^. Reemtsma and These^52^ suggested the possibility that high molecular-weight components of humic and fulvic acids were less effectively ionized by ESI than low molecular-weight components. Additionally, Piccolo and Spiteller^53^ stated that when utilizing ESI to detect DOM molecular compounds, the dominance of hydrophobic compounds in humic substances may inhibit ESI of hydrophilic compounds due to supramolecular associations of low-molecular organic compounds. However, Stenson *et al*.^54^ used ESI coupled to FT-ICR MS to detect molecular compounds in Suwannee River fulvic acids and reported that ions generated by ESI were partially representative of the entire humic substances studied. In any case, our results suggest that DOM molecular diversity does not increase or decrease with DOC concentration in water samples collected from boreal forests.

This study presents preliminary but unprecedented results on the changes in the molecular diversity and composition of DOM across water movement in a boreal forest. At this stage, we could not examine temporal variations in the quality of DOM. However, common DOM molecular compounds were detected in the same location between May and June and the number of the lignins common to both May and June was larger than that of any other biomolecular class for any water sample (Fig. S3). Therefore, it is possible that lignins regulate not only spatial but also temporal variations in molecular diversity. Further research is needed on the temporal variations and on the factors driving the spatial and temporal variations in molecular diversity and the mechanisms of preservation and alteration of DOM molecular compounds across water movement throughout the year in boreal ecosystems.

## Methods

### Site description

This study was conducted in the Kangasvaara catchment (Fig. S4) located in eastern Finland (63°51′N, 28°58′E). The area and mean elevation of the catchment are 56 ha and 187 m a.s.l., respectively. The catchment has a perennial brook. The mean slope gradient of the catchment is 7%. From 1981 to 2000, the mean annual air temperature was 2.3 °C and precipitation was 527 mm, 35% of which was snowfall^55^. The soils in the catchment mainly consist of iron podzols (haplic podzols) and fibric histosols (peat), which have developed on stony till material. The underlying bedrock consists of gneiss granite and granodiorite. The proportion of peatland is 8% of the catchment area^56^. Most of the forests on the upland mineral soils (97%) were old-growth forests dominated by Norway spruce (*Picea abies* L. Karst.), Scots pine (*Pinus sylvestris* L.), and white and silver birch (*Betula pubescens* Ehrh. and *Betula pendula* Roth., respectively). European aspen (*Populus tremula* L.) were also found in the catchment^56^.

The experimental stands in the catchment were old-growth mixed forest (F_O_) dominated by Norway spruce and 12-year-old Scots pine plantation (F_Y_). The plantation was established after harvesting a portion of the Norway spruce stands (Fig. S4). A 50 × 50 m plot was established in each stand. Norway spruce, Scots pine, and deciduous trees accounted for 53%, 33%, and 14%, respectively, of the total stand volume of 260 m^3^ ha^–1^ in the F_O_. The field layer vegetation was dominated by dwarf shrubs (*Vaccinium vitis-idaea* L. and *V. myrtillus* L.) and the bottom layer by feather mosses (*Pleurozium schreberi* Brid. and *Hylocomium splendens* (Hedw.) B. S. & G.). The forest floor consisted of litter and mor humus. In 1996, stem-only harvesting was carried out in 35% of the catchment area and Scots pine seedlings were planted thereafter. Branches, leaves, and treetops, the diameters of which were less than 8 cm, were left on site. The field layer vegetation was dominated by grasses (*Deschampsia flexuosa* (L.) Trin.) and herbs (*Epilobium angustifolium* L.), and the bottom layer by pioneer mosses (*Dicranum* sp. and *Polytrichum* sp.) in the F_Y_. The average thickness of the overlaying mor humus layer (O-horizon), the eluvial (E) horizon and the upper illuvial (B) horizon was 5.7, 6.8 and 13.9 cm, respectively.

### Water sampling

Bulk deposition, throughfall, soil water, groundwater, and stream water were sampled in May after snowmelt and in June before the dry summer months of 2010. Bulk deposition was sampled using five open collectors (each 130.7 cm^2^) in an open area within 500 m of the experimental plots (Fig. S4). Throughfall was sampled using 20 collectors of the same type used for bulk deposition, placed systematically around the F_O_ plots. The bulk deposition and throughfall samples were collected for a week in each month and emptied after the week.

The soil water was collected under the O-horizon, at a depth of 15 cm that was comparable to the E-horizon and at a depth of 35 cm that was comparable to the B-horizon by tension (60 kPa) lysimeters that consisted of 67-mm-long porous cups with an outer diameter of 12 mm (P80, Hoechst CeramTec AG, Germany). Three lysimeters (L1, L2, and L3) were placed 1–2 m apart in every horizon and each stand and soil water from each location was collected for the same week as bulk deposition and throughfall. In May, no water samples were obtained from two lysimeters in the E- and B-horizons in F_O_, one lysimeter in the O-horizon, three lysimeters in the E-horizon, and two lysimeters in the B-horizon in F_Y_ because of small rainfall. In June, no water samples were obtained from three lysimeters in the O-horizon in F_O_, three lysimeters in the O-horizon and one lysimeter in the B-horizon in F_Y_.

The groundwater was obtained from a groundwater tube installed at a depth of 0.7 m in the peat layer (groundwater-PT), 2.5 m in the moraine (groundwater-MO), and 2.4 m in the mineral soil under the peat layer (groundwater-MI). The depth of the peat layer around the groundwater-PT ranged from 1.5 to 2.1 m. The groundwater tube was made of polyamide and had an inner diameter of 30 mm and an outer diameter of 40 mm. The stream water samples were collected at the outlet of the Kangasvaara catchment (Fig. S4). The groundwater and stream water samples were taken at the same day as other water samples, where temperature, rainfall, and 10-day antecedent rainfall were 8.1 °C in May and 13.5 °C in June, 0.8 mm in May and 0 mm in June, and 27.3 mm in May and 57.3 mm in June, respectively.

All samples were filtered using pre-combusted glass fiber filters with a nominal pore size of 0.7 μm (GF/F, Whatman, Japan) and frozen until analysis.

### Chemical and data analyses

DOC concentrations were measured using the combustion catalytic oxidation method (TOC-5000; SHIMADZU Corp., Japan). DOM molecular compounds were analyzed using electrospray ionization (ESI) coupled to high-resolution FT-ICR MS. In this study, an FT-ICR mass spectrometer with a 9.4-T magnetic field was used (APEX-Q-94e, Bruker Daltonics Inc., MA, USA). Filtered samples were treated with the C_18_ solid phase extraction (SPE) method to remove inorganic salts^57^. Water samples extracted by this method were diluted with deionized water and methanol to yield a final sample composition of 50/50 (v/v) of methanol to water. The extraction efficiency ranged from 32% to 64%. The ionization efficiency was enhanced by adding ammonium hydroxide before ESI^23^. Samples were injected into the FT-ICR mass spectrometer using a syringe pump at an infusion rate of 100 μl h^–1^. All samples were analyzed in the negative ion mode. The electrospray voltage was optimized for each sample. Ions were accumulated in a hexapole for 2 s before they were transferred to the ICR cell, and the 100 transients collected using a 2 M Word time domain were co-added. All spectra (Fig. S5) were externally calibrated using an arginine standard and internally calibrated using fatty acids. Mass lists were produced using a signal-to-noise ratio (S/N) cut-off of 4. Isotope peaks were removed from the list. The molecular formula calculator (Molecular Formula Calculator ver. 1.0; ©NHMFL, 1998) was used to assign an expected molecular formula for each m/z value with a mass accuracy ≤1 ppm^23^,^27^. Only m/z values in the range of 180–500 were inserted into the molecular formula calculator. The following conditions were used for formula assignment: C = 0–∞; H = 0–∞; O = 0–∞; N = 0–5; S = 0–3; P = 0–3; DBE ≥ 0^27^. After the formula assignment by the molecular formula calculator, some formulas not likely to be observed in natural water were eliminated based on rules described in Kujawinski and Behn^58^ and Wozniak *et al*.^34^.

To identify which biomolecular class each molecular compound belonged to, a van Krevelen diagram was used based on the elemental ratios of the expected molecular formulas, i.e., the oxygen-to-carbon (O/C) and hydrogen-to-carbon ratios (H/C)^26^ (Fig. S6). Here, we excluded the molecules that had multi-candidate formulas assigned and thus did not have a particular range of O/C and H/C values that falls into a biomolecular class. The excluded molecules accounted for 40% of the total m/z peaks on average, with variability ranging from 19% to 55%, and tended to increase with increasing molecular weight. Following the protocols proposed by Grannas *et al*.^27^ and Hockaday *et al*.^28^, each molecular compound was divided into seven biomolecular classes based on a certain range of O/C and H/C values. These included lipids: H/C > 1.5, 0 ≤ O/C < 0.3; proteins: H/C > 1.5, 0.3 ≤ O/C ≤ 0.67; aminosugars/carbohydrates (As/Ch): H/C > 1.5, O/C > 0.67; unsaturated hydrocarbons (UH): 0.7 ≤ H/C ≤ 1.5, O/C < 0.1; lignin-like molecules (lignins): 0.7 ≤ H/C ≤ 1.5, 0.1 ≤ O/C ≤ 0.67; tannin-like molecules (tannins): 0.5 ≤ H/C ≤ 1.5, 0.67 < O/C; and condensed aromatic structures (CAS): 0.2 ≤ H/C < 0.7, O/C ≤ 0.67.

DOC concentration and the number of molecular compounds detected were compared among sampling locations using a one-way ANOVA. The number of replicates ranged from 2 to 4 at each location. Tukey-Kramer post-hoc tests were used to compare sample means. Normality (Shapiro-Wilk test) and homogeneity (Bartlett test) were verified in advance. Jaccard similarity coefficients were calculated to examine how many molecular compounds were the same between different sampling locations, where coefficient = 1 indicates that two water samples share all of the molecular compounds while coefficient = 0 indicates that there are no common molecular compounds. Then, dendrograms of different sampling locations were obtained for all molecular compounds detectable by FT-ICR MS and for each biomolecular class by calculating the Jaccard’s distance between samples and subsequently using Ward’s method. Additionally, correlation coefficients were calculated between cophenetic matrices of Jaccard’s distance for all molecular compounds and for each biomolecular class to examine which biomolecular class dendrogram exhibits a similar pattern to the dendrogram for all molecular compounds. Statistical analyses in this study were conducted using R (version 2.13.1^59^).

## Additional Information

**How to cite this article:** Ide, J. *et al*. Spatial variations in the molecular diversity of dissolved organic matter in water moving through a boreal forest in eastern Finland. *Sci. Rep.* **7**, 42102; doi: 10.1038/srep42102 (2017).

**Publisher's note:** Springer Nature remains neutral with regard to jurisdictional claims in published maps and institutional affiliations.

## Supplementary Material

Supplementary Information

## References

[b1] Oliver B. G., Thurman E. M. & Malcolm R. L. The contribution of humic substances to the acidity of colored natural waters. Geochim. Cosmochim. Acta 47, 2031–2035 (1983).

[b2] Meili M. Sources, concentrations and characteristics of organic matter in softwater lakes and streams of the Swedish forest region. Hydrobiologia 229, 23–41 (1992).

[b3] Kortelainen P. Content of Total Organic Carbon in Finnish Lakes and Its Relationship to Catchment Characteristics. Can. J. Fish. Aquat. Sci. 50, 1477–1483 (1993).

[b4] Green S. A. & Blough N. V. Optical absorption and fluorescence properties of chromophoric dissolved organic matter in natural waters. Limnol. Oceanogr. 39, 1903–1916 (1994).

[b5] Chiwa M., Crossley A., Sheppard L. J., Sakugawa H. & Cape J. N. Throughfall chemistry and canopy interactions in a Sitka spruce plantation sprayed with six different simulated polluted mist treatments. Environ. Pollut. 127, 57–64 (2004). 14553995 10.1016/s0269-7491(03)00259-8

[b6] Koenings J. P. & Hooper F. F. The influence of colloidal organic matter on iron and iron-phosphorus cycling in an acid bog lake. Limnol. Oceanogr. 21, 684–696 (1976).

[b7] Francko D. A. Epilimnetic Phosphorus Cycling: Influence of Humic Materials and Iron on Coexisting Major Mechanisms. Can. J. Fish. Aquat. Sci. 43, 302–310 (1986).

[b8] Jones R. I., Salonen K. & Haan H. Phosphorus transformations in the epilimnion of humic lakes: abiotic interactions between dissolved humic materials and phosphate. Freshw. Biol. 19, 357–369 (1988).

[b9] Mierle G. & Ingram R. The role of humic substances in the mobilization of mercury from watersheds. Water Air Soil Pollut. 56, 349–357 (1991).

[b10] Winch S., Ridal J. & Lean D. Increased Metal Bioavailability Following Alteration of Freshwater Dissolved Organic Carbon by Ultraviolet B Radiation Exposure. Environ. Toxicol. 17, 267–274 (2002). 12112635 10.1002/tox.10057

[b11] Qualls R. G. & Haines B. L. Biodegradability of Dissolved Organic Matter in Forest Throughfall, Soil Solution, and Stream Water. Soil Sci. Soc. Am. J. 56, 578–586 (1992).

[b12] Ward N. D. . Degradation of terrestrially derived macromolecules in the Amazon River. Nat. Geosci. 6, 530–533 (2013).

[b13] Dixon R. K. . Carbon pools and flux of global forest ecosystems. Science 263, 185–90 (1994). 17839174 10.1126/science.263.5144.185

[b14] Callesen I. . Soil carbon stores in Nordic well-drained forest soils-relationships with climate and texture class. Glob. Chang. Biol. 9, 358–370 (2003).

[b15] Piirainen S., Finér L., Mannerkoski H. & Starr M. Effects of forest clear-cutting on the carbon and nitrogen fluxes through podzolic soil horizons. Plant Soil 239, 301–311 (2002).

[b16] Fröberg M., Berggren D., Bergkvist B., Bryant C. & Mulder J. Concentration and Fluxes of Dissolved Organic Carbon (DOC) in Three Norway Spruce Stands along a Climatic Gradient in Sweden. Biogeochemistry 77, 1–23 (2006).

[b17] Kothawala D. N., Moore T. R. & Hendershot W. H. Soil Properties Controlling the Adsorption of Dissolved Organic Carbon to Mineral Soils. Soil Sci. Soc. Am. J. 73, 1831–1842 (2009).

[b18] Kaiser K., Guggenberger G., Haumaier L. & Zech W. Seasonal variations in the chemical composition of dissolved organic matter in organic forest floor layer leachates of old-growth Scots pine (*Pinus sylvestris* L.) and European beech (*Fagus sylvatica* L.) stands in northeastern Bavaria, Germany. Biogeochemistry 55, 103–143 (2001).

[b19] Piirainen S., Finér L., Mannerkoski H. & Starr M. Carbon, nitrogen and phosphorus leaching after site preparation at a boreal forest clear-cut area. For. Ecol. Manage. 243, 10–18 (2007).

[b20] Bourbonniere R. A. & Creed I. F. Biodegradability of dissolved organic matter extracted from a chronosequence of forest-floor materials. J. Plant Nutr. Soil Sci. 169, 101–107 (2006).

[b21] Qualls R. G., Haines B. L. & Swank W. T. Fluxes of Dissolved Organic Nutrients and Humic Substances in a Deciduous Forest. Ecology 72, 254 (1991).

[b22] Sleighter R. L. & Hatcher P. G. The application of electrospray ionization coupled to ultrahigh resolution mass spectrometry for the molecular characterization of natural organic matter. J. Mass Spectrom. 42, 559–574 (2007). 17474116 10.1002/jms.1221

[b23] Sleighter R. L., McKee G. a., Liu Z. & Hatcher P. G. Naturally present fatty acids as internal calibrants for Fourier transform mass spectra of dissolved organic matter. Limnol. Oceanogr. Methods 6, 246–253 (2008).

[b24] Sleighter R. L. . Establishing a Measure of Reproducibility of Ultrahigh-Resolution Mass Spectra for Complex Mixtures of Natural Organic Matter. Anal. Chem. 84, 9184–9191 (2012). 23075323 10.1021/ac3018026

[b25] Kido Soule M. C., Longnecker K., Giovannoni S. J. & Kujawinski E. B. Impact of instrument and experiment parameters on reproducibility of ultrahigh resolution ESI FT-ICR mass spectra of natural organic matter. Org. Geochem. 41, 725–733 (2010).

[b26] van Krevelen D. W. Graphical-statistical method for the study of structure and reaction processes of coal. Fuel 29, 269–284 (1950).

[b27] Grannas A. M., Hockaday W. C., Hatcher P. G., Thompson L. G. & Mosley-Thompson E. New revelations on the nature of organic matter in ice cores. J. Geophys. Res. 111, D04304 (2006).

[b28] Hockaday W. C., Purcell J. M., Marshall A. G., Baldock J. A. & Hatcher P. G. Electrospray and photoionization mass spectrometry for the characterization of organic matter in natural waters: a qualitative assessment. Limnol. Oceanogr. Methods 7, 81–95 (2009).

[b29] Kalbitz K. . Changes in properties of soil-derived dissolved organic matter induced by biodegradation. Soil Biol. Biochem. 35, 1129–1142 (2003).

[b30] Nebbioso A. & Piccolo A. Molecular characterization of dissolved organic matter (DOM): a critical review. Anal. Bioanal. Chem. 405, 109–124 (2013). 22965531 10.1007/s00216-012-6363-2

[b31] Kim S., Kramer R. W. & Hatcher P. G. Graphical Method for Analysis of Ultrahigh-Resolution Broadband Mass Spectra of Natural Organic Matter, the Van Krevelen Diagram. Anal. Chem. 75, 5336–5344 (2003). 14710810 10.1021/ac034415p

[b32] Witt M., Schmitt-Kopplin P., Hertkorn N. & Frommberger M. Analysis of complex, natural Suwannee river fulvic acid mixtures by FTMS: Revealing homologous series by analyzing ultra-high resolved mass spectra by Kendrick and van-Krevelen plots. *Proc. Int. Mass Spectrom. Conf.* (2006).

[b33] Kim S., Kaplan L. A. & Hatcher P. G. Biodegradable dissolved organic matter in a temperate and a tropical stream determined from ultra-high resolution mass spectrometry. Limnol. Oceanogr. 51, 1054–1063 (2006).

[b34] Wozniak A. S., Bauer J. E., Sleighter R. L., Dickhut R. M. & Hatcher P. G. Technical Note: Molecular characterization of aerosol-derived water soluble organic carbon using ultrahigh resolution electrospray ionization Fourier transform ion cyclotron resonance mass spectrometry. Atmos. Chem. Phys. 8, 5099–5111 (2008).

[b35] Mazzoleni L. R., Ehrmann B. M., Shen X., Marshall A. G. & Collett J. L. Water-Soluble Atmospheric Organic Matter in Fog: Exact Masses and Chemical Formula Identification by Ultrahigh-Resolution Fourier Transform Ion Cyclotron Resonance Mass Spectrometry. Environ. Sci. Technol. 44, 3690–3697 (2010). 20397689 10.1021/es903409k

[b36] Guggenberger G. & Zech W. Composition and dynamics of dissolved carbohydrates and lignin-degradation products in two coniferous forests, N.E. Bavaria, Germany. Soil Biol. Biochem. 26, 19–27 (1994).

[b37] Minor E. C., Steinbring C. J., Longnecker K. & Kujawinski E. B. Characterization of dissolved organic matter in Lake Superior and its watershed using ultrahigh resolution mass spectrometry. Org. Geochem. 43, 1–11 (2012).

[b38] Wickland K. P., Neff J. C. & Aiken G. R. Dissolved Organic Carbon in Alaskan Boreal Forest: Sources, Chemical Characteristics, and Biodegradability. Ecosystems 10, 1323–1340 (2007).

[b39] Hur J., Park M.-H. & Schlautman M. A. Microbial Transformation of Dissolved Leaf Litter Organic Matter and Its Effects on Selected Organic Matter Operational Descriptors. Environ. Sci. Technol. 43, 2315–2321 (2009). 19452880 10.1021/es802773b

[b40] Kawasaki M., Ohte N. & Katsuyama M. Biogeochemical and hydrological controls on carbon export from a forested catchment in central Japan. Ecol. Res. 20, 347–358 (2005).

[b41] Martin J. P., Haider K. & Kassim G. Biodegradation and Stabilization after 2 Years of Specific Crop, Lignin, and Polysaccharide Carbons in Soils. Soil Sci. Soc. Am. J. 44, 1250 (1980).

[b42] Haider K. Problems related to the humification processes in soils of temperate climates in Soil biochemistry 7 (eds. Stotzky, G. & Bollag, J.-M.) 55–94 (CRC Press, 1992).

[b43] Dawson H. J., Ugolini F. C., Hrutfiord B. F. & Zachara J. Role of soluble organics in the soil processes of a podozol, central cascades, Washington. Soil Sci. 126, 290–296 (1978).

[b44] Cronan C. S. & Aiken G. R. Chemistry and transport of soluble humic substances in forested watersheds of the Adirondack Park, New York. Geochim. Cosmochim. Acta 49, 1697–1705 (1985).

[b45] Lundström U., van Breemen N. & Bain D. The podzolization process. A review. Geoderma 94, 91–107 (2000).

[b46] Sleighter R. L. & Hatcher P. G. Molecular characterization of dissolved organic matter (DOM) along a river to ocean transect of the lower Chesapeake Bay by ultrahigh resolution electrospray ionization Fourier transform ion cyclotron resonance mass spectrometry. Mar. Chem. 110, 140–152 (2008).

[b47] Reemtsma T. Determination of molecular formulas of natural organic matter molecules by (ultra-) high-resolution mass spectrometry. J. Chromatogr. A 1216, 3687–3701 (2009). 19264312 10.1016/j.chroma.2009.02.033

[b48] Boerjan W., Ralph J. & Baucher M. Lignin Biosynthesis. Annu. Rev. Plant Biol. 54, 519–546 (2003). 14503002 10.1146/annurev.arplant.54.031902.134938

[b49] Kalbitz K., Solinger S., Park J.-H., Michalzik B. & Matzner E. Controls on the dynamics of dissolved organic matter in soils: a review. Soil Sci. 165, 277–304 (2000).

[b50] Don A. & Kalbitz K. Amounts and degradability of dissolved organic carbon from foliar litter at different decomposition stages. Soil Biol. Biochem. 37, 2171–2179 (2005).

[b51] Kujawinski E. B., Del Vecchio R., Blough N. V., Klein G. C. & Marshall A. G. Probing molecular-level transformations of dissolved organic matter: Insights on photochemical degradation and protozoan modification of DOM from electrospray ionization Fourier transform ion cyclotron resonance mass spectrometry. Mar. Chem. 92, 23–37 (2004).

[b52] Reemtsma T. & These A. On-line coupling of size exclusion chromatography with electrospray ionisation - tandem mass spectrometry for the analysis of aquatic fulvic and humic acids. Anal. Chem. 75, 1500–1507 (2003). 12659215 10.1021/ac0261294

[b53] Piccolo A. & Spiteller M. Electrospray ionization mass spectrometry of terrestrial humic substances and their size fractions. Anal. Bioanal. Chem. 377, 1047–59 (2003). 12955391 10.1007/s00216-003-2186-5

[b54] Stenson A. C., Marshall A. G. & Cooper W. T. Exact Masses and Chemical Formulas of Individual Suwannee River Fulvic Acids from Ultrahigh Resolution Electrospray Ionization Fourier Transform Ion Cyclotron Resonance Mass Spectra. Anal. Chem. 75, 1275–1284 (2003). 12659186 10.1021/ac026106p

[b55] Palviainen M. . Nitrogen, Phosphorus, Carbon, and Suspended Solids Loads from Forest Clear-Cutting and Site Preparation: Long-Term Paired Catchment Studies from Eastern Finland. Ambio 43, 218–233 (2014). 24046144 10.1007/s13280-013-0439-x PMC3906482

[b56] Finér L. . In Effects of Harvesting and Scarification on Water and Nutrient Fluxes: A Description of Catchments and Methods, and Results from the Pretreatment Calibration Period. The Finnish Forest Research Institute, Research Papers *648* (1997).

[b57] Kim S., Simpson A. J., Kujawinski E. B., Freitas M. A. & Hatcher P. G. High resolution electrospray ionization mass spectrometry and 2D solution NMR for the analysis of DOM extracted by C18 solid phase disk. Org. Geochem. 34, 1325–1335 (2003).

[b58] Kujawinski E. B. & Behn M. D. Automated Analysis of Electrospray Ionization Fourier Transform Ion Cyclotron Resonance Mass Spectra of Natural Organic Matter. Anal. Chem. 78, 4363–4373 (2006). 16808443 10.1021/ac0600306

[b59] R Development Core Team, R. R: A Language and Environment for Statistical Computing. R Foundation for Statistical Computing, (R Foundation for Statistical Computing, 2011).

