# Supplementary material for: Spatial variations in the molecular diversity of dissolved organic matter in water moving through a boreal forest in eastern Finland
Source: Sci Rep. 2017 Feb 10;7:42102. doi: 10.1038/srep42102 (PMC5301308; doi:10.1038/srep42102)
Supplement: Supplementary Information [file srep42102-s1.pdf]

Supplementary Information

Spatial variations in the molecular diversity of dissolved organic matter in water moving through a boreal forest in eastern Finland

Jun'ichiro Ide[a]*, Mizue Ohashi[b], Katsutoshi Takahashi[c], Yuko Sugiyama[b], Sirpa Piirainen[d], Pirkko Kortelainen[e], Nobuhide Fujitake[f], Keitaro Yamase[g], Nobuhito Ohte[h], Mina Moritani[b], Miyako Hara[b], Leena Finér[d]

[a]Institute of Decision Science for a Sustainable Society, Kyushu University, Fukuoka 811-2415, Japan

[b]Graduate School of Human Science and Environment, University of Hyogo, Hyogo 670-0092, Japan

[c]National Institute of Advanced Industrial Science and Technology, Tsukuba 305-8568, Japan

[d]Natural Resources Institute Finland, P.O. Box 68, FIN-80101 Joensuu, Finland

[e]Finnish Environment Institute, P.O. Box 140, FIN-00251 Helsinki, Finland

[f]Faculty of Agriculture, Kobe University, Kobe 657-0013, Japan

[g]Hyogo Prefectural Technology Center for Agriculture, Forestry and Fisheries, Forestry and Forest Products Research Institute, Hyogo 671-2515, Japan

[h]Department of Social Informatics, Graduate School of Informatics, Kyoto University, Kyoto 606-8501, Japan

***Corresponding author:**

Institute of Decision Science for a Sustainable Society, Kyushu University, 394 Tsubakuro, Sasaguri, Fukuoka 811-2415, Japan.

Tel.: +81-(0)92-948-3109, E-mail: ide.junichiro@gmail.com

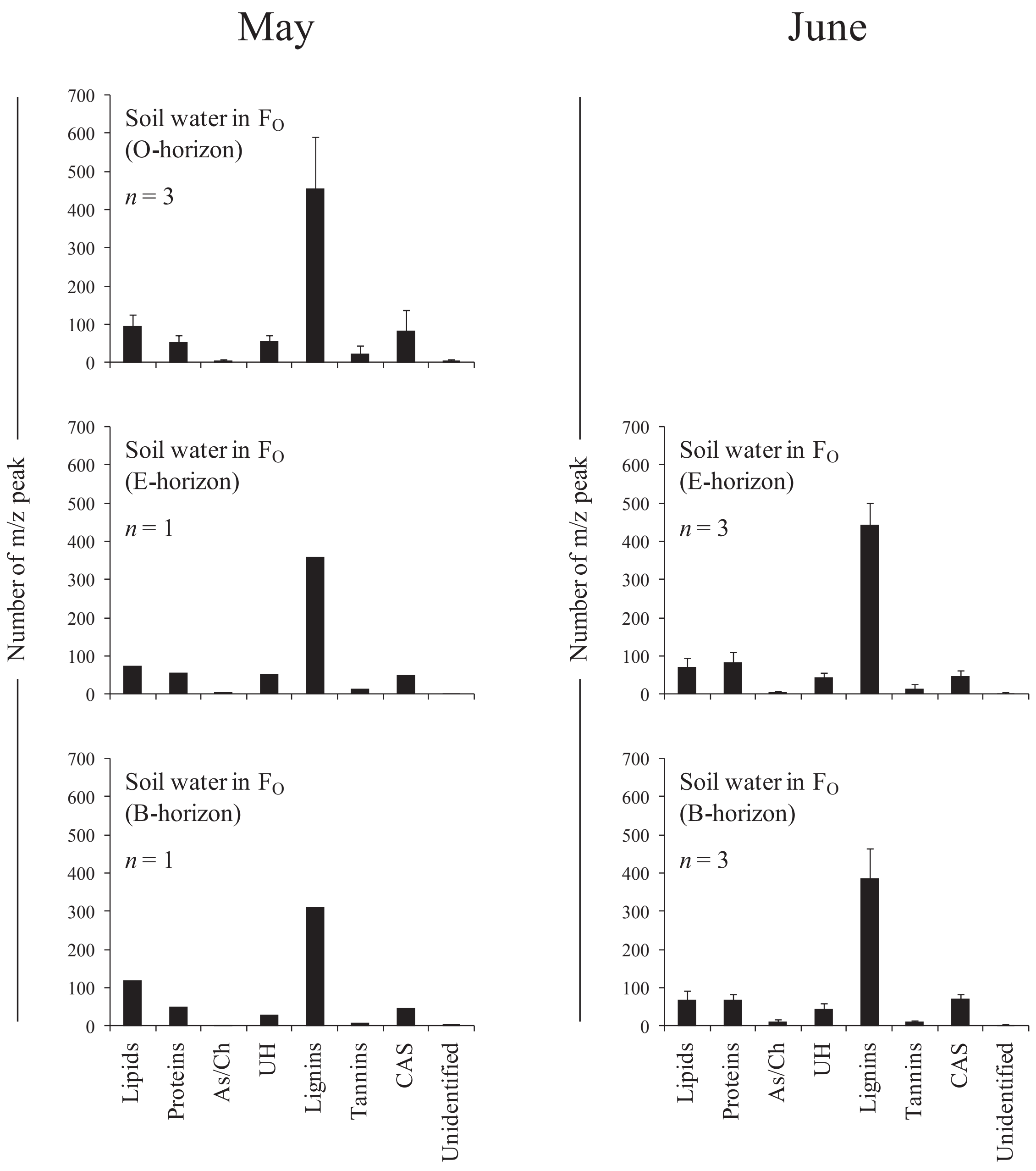

Figure S1 Classification of the m/z peaks of soil water in an old-growth forest (F_O) into specific biomolecular classes using a van Krevelen diagram. Error bars represent standard deviations. See Methods for the abbreviations of biomolecular classes.

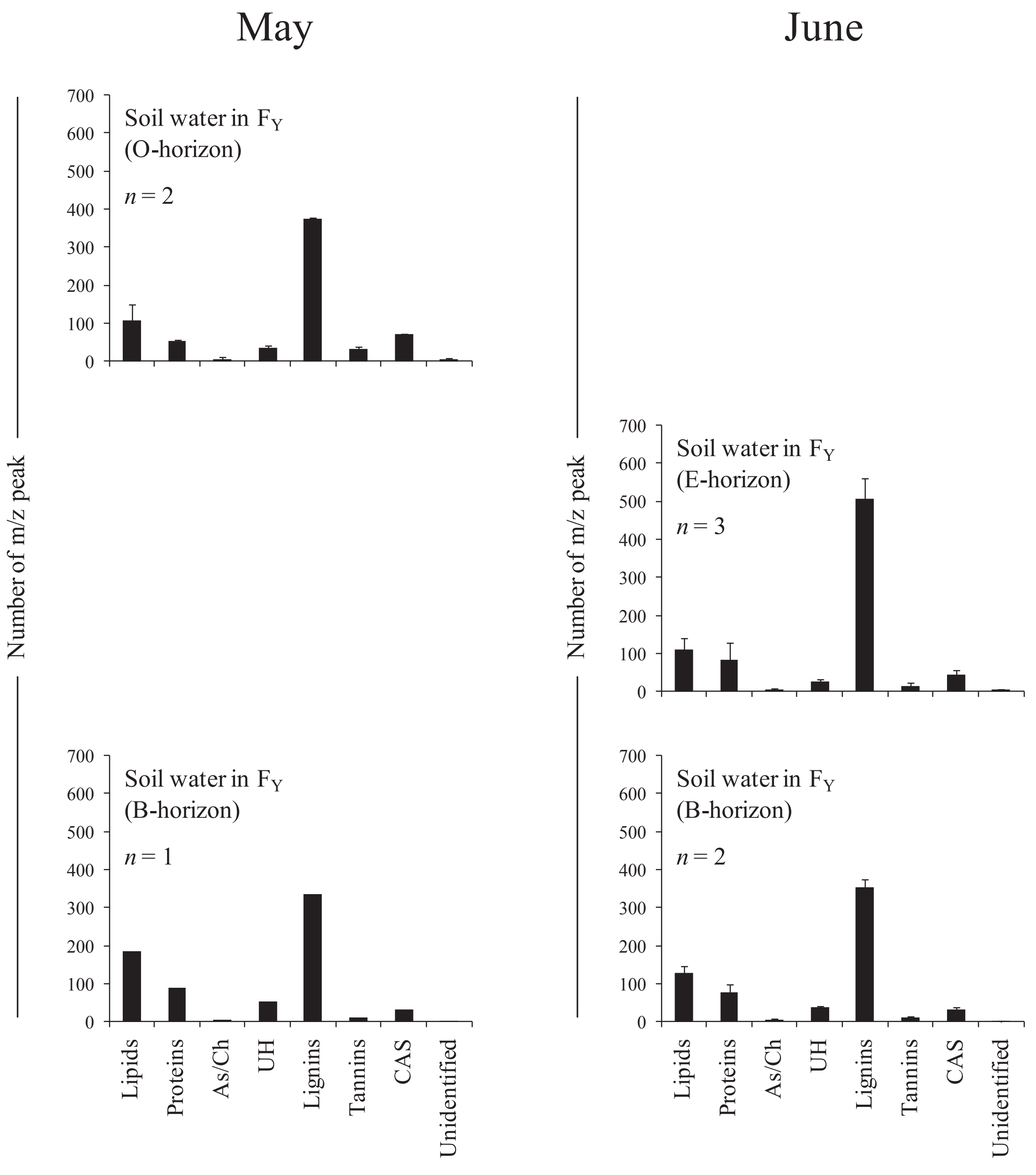

Figure S2 Classification of the m/z peaks of soil water sample in the young plantation (F_Y) into specific biomolecular classes using a van Krevelen diagram. Error bars represent standard deviations. See Methods for the abbreviations of biomolecular classes.

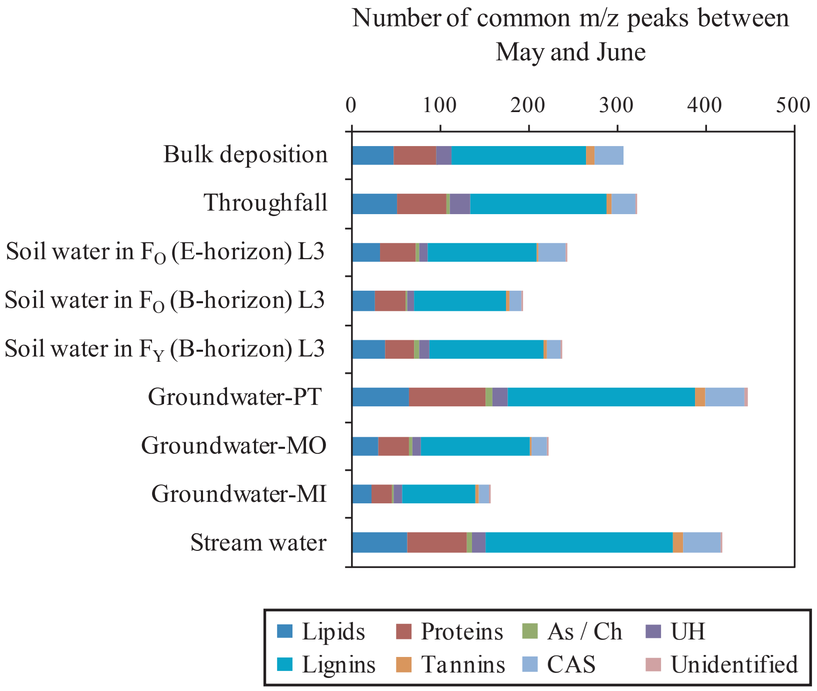

Figure S3 The number of common m/z peaks classified into specific biomolecular classes between May and June at the same sampling locations. F_O and F_Y represent old-growth forest and young plantation stands, respectively. See Methods for the abbreviations of groundwater and biomolecular classes.

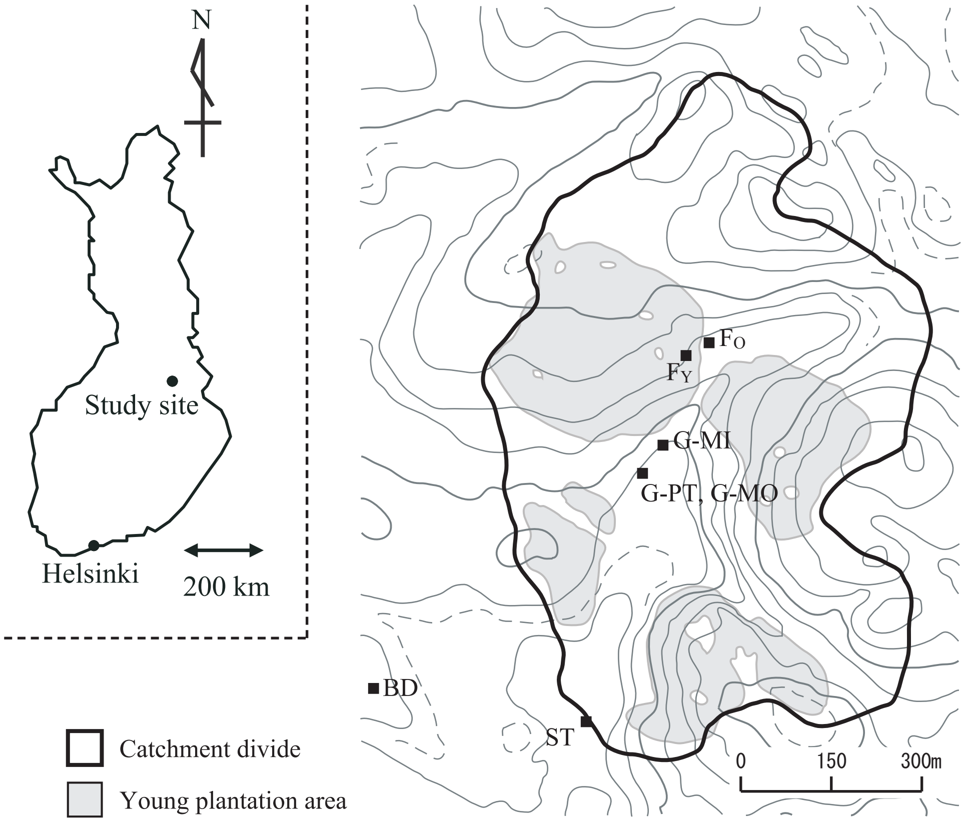

Figure S4 Map of the Kangasvaara catchment and water sampling locations. F_O and F_Y represent old-growth forest and young plantation stands, respectively, the site at which soil water was sampled. BD and ST represent the sampling locations of bulk deposition and stream water, respectively. G-PT, G-MO, and G-MI represent the sampling locations of groundwater within the peat layer, moraine, and mineral soils under the peat layer, respectively. The map was generated using Adobe Illustrator (ver. 11.0.0) based on ArcMap (ver. 9.3).

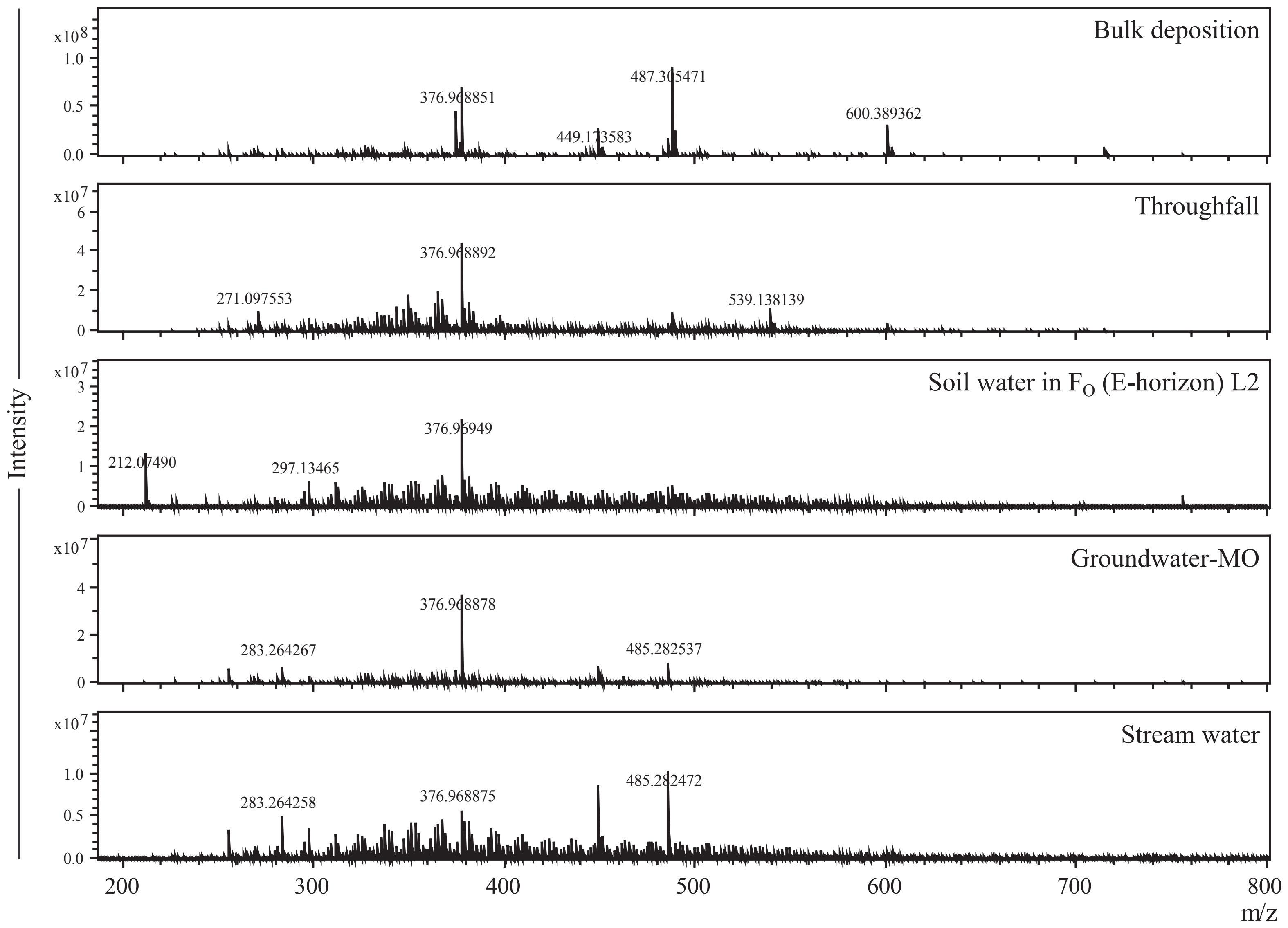

Figure S5 Mass spectra of dissolved organic matter (DOM) samples for bulk deposition, throughfall, E-horizon soil water in an old-growth forest (F_O), groundwater within the moraine (MO), and stream water collected in June 2010 as examples of the FT-ICR mass spectrum.

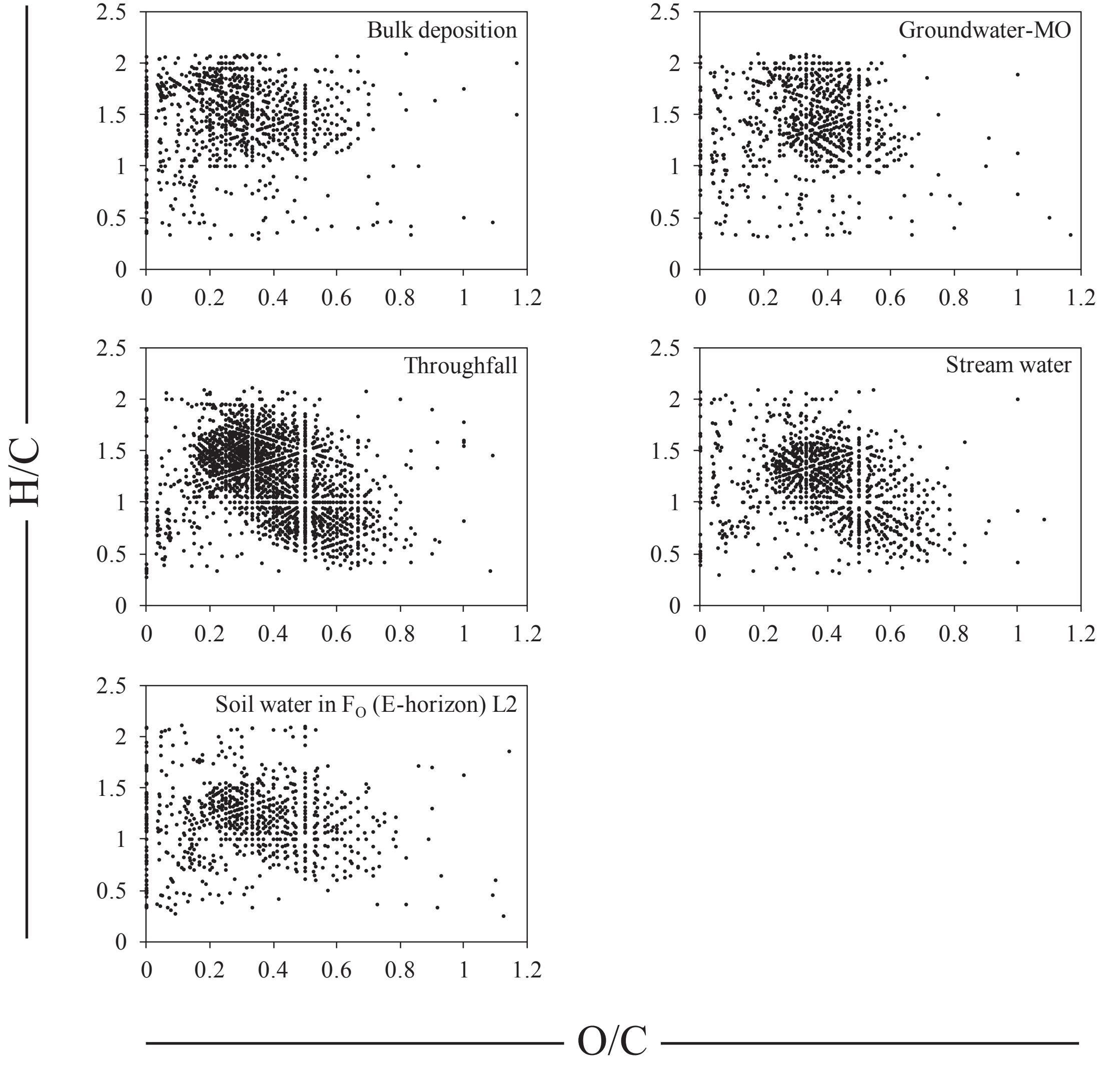

Figure S6 Molecular element ratio plots of dissolved organic matter (DOM) samples for bulk deposition, throughfall, E-horizon soil water in an old-growth forest (F_O), groundwater within the moraine (MO), and stream water collected in June 2010 as examples of the van Krevelen diagram.
